# Influence of Marangoni Effect on Heat and Mass Transfer during Evaporation of Sessile Microdroplets

**DOI:** 10.3390/mi13111968

**Published:** 2022-11-13

**Authors:** Haiting Liu, Jiewen Deng

**Affiliations:** School of Energy and Power Engineering, Northeast Electric Power University, Jilin 132012, China

**Keywords:** droplet evaporation, Marangoni effect, thermal mass coupling, enhanced evaporation, computational fluid dynamics

## Abstract

Evaporative cooling is an important method for controlling the temperature of micro devices, and heat and mass transfer from the microdroplets in the evaporation process directly affect the cooling performance. In order to study the droplet heat and mass transfer law in the droplet evaporation process, this paper builds a coupled thermal mass model of droplet evaporation and tests the accuracy of the numerical model through theoretical results. In order to study the influence of the Marangoni effect on the droplet evaporation process and the effects of different initial droplet radius and ambient temperature on the temperature and flow, fields within the droplet are compared. From this result, it can be seen that the droplet volume is 20 μL, and the maximum flow velocity in the droplet is 0.34 mm/s, without taking into account the Marangoni effect. When the Marangoni effect is taken into account, the maximum flow velocity increases by almost 100 times. The Marangoni effect can cause the convection in the droplet to change direction, and the formation of the Marangoni flow may affect the temperature distribution within the droplet, thereby increasing the evaporation efficiency by 2.5%. The evaporation process will increase the velocity of the air close to the surface of the liquid, but the increase in air velocity close to the liquid surface is not sufficient to reinforce evaporation. There is a non-linear relationship between increasing ambient temperature and increasing evaporation efficiency. For every 5 °C increase in ambient temperature, the maximum increase in the rate of evaporation is approximately 22.7%.

## 1. Introduction

Droplet evaporation is common in nature and human life. In practical engineering applications, many industrial technologies are closely related to droplet evaporation—such as inkjet printing, desalination of seawater, concentration of waste fluid, cooling of working fluids and medical diagnostics—and typically occur spontaneously when the vapor pressure near the liquid-gas interface of a liquid droplet is not saturated. This is a complex physical process accompanied by convective mass transfer and heat transfer accompanied by a phase change.

In 1805, following the landmark work on liquid evaporation by Young [1], one popular topic has become the process of evaporation-induced intra-drop changes. Researchers studying evaporation have uncovered many interesting phenomena caused by the process of evaporation, such as motion between liquid and solid during evaporation, heat transfer between droplet and substrate, and nanoparticle effect on evaporation. Marangoni found that changes in the concentration of local constituents on the surface of the liquid can lead to changes in surface tension, causing the fluid to flow out of a position with a lower surface tension and into a position with a higher surface tension [2]. This type of liquid flow due to gradients in surface tension has since been referred to as the “Marangoni effect”. Gibbs [3] provided a more detailed and seminal theoretical contribution to this phenomenon, and thus, it is also called the “Gibbs-Marangoni effect”. While research is ongoing, a large body of work has demonstrated that there is a link between liquid surface tension and droplet evaporation [4,5,6]. In order to test the effect of gravity on evaporation in the binary solution evaporation process, a mathematical model was proposed by Diddens [7] for predicting Rayleigh and Maranni convection phenomena. These results demonstrate that, while gravity may play a role in promoting evaporation, natural convection is the primary driving mechanism for the internal flow of binary droplets. The distribution law of the temperature and velocity fields under the Marangoni action was obtained by Girard [8]. By constructing averages of numerical simulations, he studied the influence of the Marangoni effect on evaporation and believed that heat conduction within the droplet was the major factor affecting the rate of evaporation.

The Marangoni effect is beneficial for improving the efficiency of micro-scale thermal mass transport and has an important influence on the component mass transfer process— not only in chemical processes, such as extraction and distillation [9], but also in heat transfer processes, such as evaporation [10] and condensation [11], which play an important role in increasing heat transfer within liquid droplets and films. Increased evaporation of droplets attached to the wall is also an important branch of research. The quasi-stationary Marangoni contraction of binary mixtures was studied by Stefan [12] through experiments and numerical analysis, and Stefan related it to the hydrodynamic model of droplet evaporation and compared the experimental results with numerical simulation. We propose that there is a general power–law relationship between the quasi-expansion contact angle and the relative saturation of the ambient gas phase. Marcus [13] distributed the fluid as an array of droplets on a super hydrophobic dose plate with 3 × 3 array pores, and the microheaters were screen printed on the back of the dose plate to study droplet evaporation on these pores; accurate measurement of the microdroplets is achieved. Ye [14] carried out an experimental study on the steady state evaporation of isopropanol droplets at different substrate temperatures. This study demonstrates that the height of the droplet can affect the evolution of the droplet flow pattern during the evaporation process, and increasing substrate temperature can cause droplet number of internal wrinkles. Wang [15] established a model for droplet evaporation on high-temperature substrates and droplet evaporation in suspension in air at elevated temperature and investigated the steady state flow process of Marangoni convection within droplets during evaporation. A numerical simulation was performed to investigate the effect of contact angle on Marangoni convection. Hu [16] constructed a relatively complete model of evaporation and investigated the characteristics of evaporation on both heated and unheated substrates, cooling effect. In addition, the theoretical model also considers buoyancy-driven convection and Marangoni convection. On the other hand, many simulations of evaporating droplet properties and inducing factors have been performed by many authors [17,18,19,20] in order to obtain the flow characteristics of Marangoni convection within the droplet on a tilted substrate, both in the oil phase and under laser irradiation, while also considering their influence on the heat and mass transfer process.

In general, the velocity of the liquid surface airflow is also a major factor affecting the evaporation efficiency, and the process of droplet evaporation increases the rate of liquid surface flow. It is, therefore, important to study whether droplet evaporation is a self-reinforcing process and to understand the effect of the Marangoni effect on evaporation; however, these studies have not discussed these issues but have mainly focused on the effects of the contact angle of the droplet and the temperature of the substrate on Marangoni convection.

The main purpose of this paper is to compare the heat and mass transfer differences in the wall droplet evaporation process with and without the Marangoni effect. The influence of ambient temperature and initial droplet size on evaporation under the influence of the Marangoni effect is also analyzed; the contribution of Marangoni convection during evaporation is analyzed in parallel.

## 2. Model Construction and Validation

### 2.1. Physical Model

**Figure 1:**
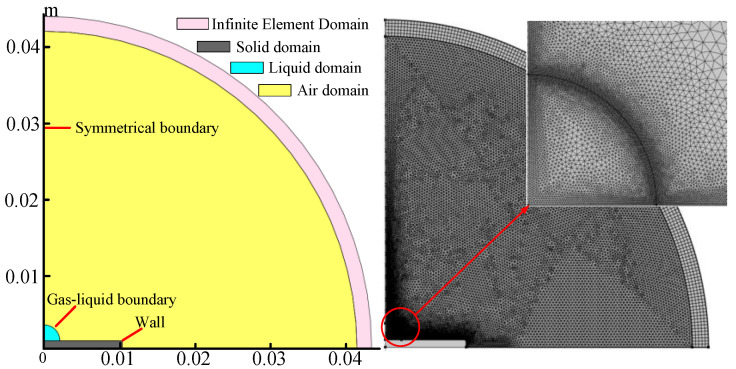
Model boundary conditions and mesh distribution.

In order to study the dynamic change process of the droplet surface during evaporation, this paper adopts the moving grid method to track the interface between the droplet and the air. This method realizes the simultaneous change of mass points and grid nodes at the moving interface so as to achieve the accurate capture of the location of the calculated interface by the deformed grid. The model boundary conditions and grid situation are shown in Figure 1. The initial radius of the droplet is *R*; the height of the stainless steel substrate is 1 mm; the width is 5*R*; the radius of the air domain is 20*R*; and the thickness of the infinite element domain is *R*. The minimum grid cell in the droplet region is 0.02 mm; the maximum grid cell is 0.08 mm; and the ambient temperature is *T_en_*.

### 2.2. Mathematical Model for the Internal Flow and Heat Transfer of Liquid Droplets

In this paper, we focus on the process of droplet evaporation on the surface of substrates. During evaporation, temperature gradients are generated within the droplet and surrounding air, which affect the convection process within the droplets and close to the liquid surface; hence, when describing the droplet evaporation process via numerical models, not only must the process of mass transfer of vapor and air around the droplet be considered but also the effect of convection caused by the temperature gradient during the evaporation process. In order to enhance the practicality of the numerical model, before constructing the numerical model in this paper, we make the following assumptions:

(1) Since some research findings show that the constant contact angle (CCA) [21] mode of evaporation accounts for over 50% of the evaporation process when the droplet is evaporating on a surface of constant heat [22,23], the process of evaporation is considered to be the CCA mode, and the change in contact angle is ignored.
(2) Droplet evaporation does not account for the change in moisture content of the initial environment, and to simplify the computational model, the environmental water vapor content is assumed to be 0 at the initial time, and water vapor in the environment only originates from the droplet during evaporation.
(3) Since the object of investigation in this paper is a micro-droplet and its size is small, the change in curvature of the liquid surface during evaporation is ignored. The effect of the curvature of the droplet interface on the concentration of vapor saturation can be neglected, according to the Kelvin equation [24]; thus, the vapor concentration at the droplet surface can be thought of as the saturated vapor concentration only relative to the local temperature, given by Equation (8).
(4) In the computational domain, the fluid is assumed to be incompressible due to the small velocity of the air flow around the droplet and the droplet.

**Figure 2:**
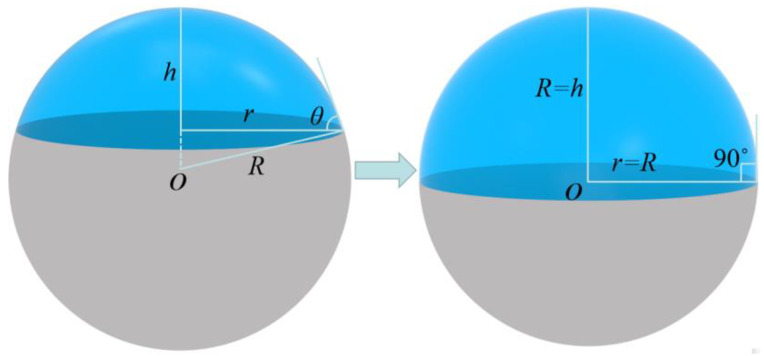
Schematic diagram of a spherical crown droplet.

Since this paper is investigating the process of droplet evaporation on a wall with a contact angle of 90°, as can be seen in Figure 2, the droplets can be equated to a spherical corona shape with the following volume equation:

(1) $$V=\pi h\left(3r^{2}+h^{2}\right)/6$$

The droplet contact angle is 90°, so the evaporation process does not take into account the change in contact angle. The volume of the droplet at which *R = r = h* and at which the droplet is hemispherical is thus:

(2) $$V=2\pi R^{3}/3$$

The velocity field *u*(*r*, *z*), the vapor concentration field *c*(*r*, *z*) and the temperature field *T*(*r*, *z*) within the droplet and in the surrounding air during the evaporation of the droplet can be described by the mass conservation equation, the momentum conservation equation and the energy conservation equation, respectively, as follows:

(3) $$\frac{\partial \rho }{\partial t}+\nabla \cdot \left(\rho u\right)=0$$

(4) $$\frac{\partial \left(\rho u\right)}{\partial t}+\rho u\cdot \nabla u=-\nabla P-\rho g+\nabla \left[\mu \left(\nabla u+{\left(\nabla u\right)}^{T}\right)-\frac{2}{3}\mu \left(\nabla \cdot u\right)\right]$$

(5) $$\frac{\partial \left(\rho c_{p}T\right)}{\partial t}+\nabla \cdot \left(\rho c_{p}uT\right)=\nabla \cdot \left(k\nabla T\right)$$

where *u* is the fluid velocity vector. *P* is the pressure; *μ* is the kinetic viscosity; and *ρ* is the fluid density, which is a temperature-dependent function, described as follows:

(6) $$\left\{\begin{array}{c} \rho_{liquid}=\rho_{0}\left[1+\alpha \left(T-T_{0}\right)\right]\\ \rho_{air}=\frac{P_{air}}{RT}M_{air}+\frac{M_{vapor}-M_{air}}{M_{vapor}}c \end{array}\right.$$

where *P_air_* is the dry air pressure; *R* is the universal gas constant; *M_vapor_* and *M_air_* are the molar masses of water vapor and dry air, respectively; and *c* is the water vapor mass concentration.

The saturation vapor pressure is derived from the Antoine equation:

(7) $$P_{sat\;}=10^{\left[A-B/\left(C+T\right)\right]}$$

where *A*, *B* and *C* use the constants in the literature [25] and take the values 8.10765, 1750.286 and 235, respectively. The saturated vapor concentration is obtained from the ideal gas law as:

(8) $$c_{sat\;}=\frac{M_{vapor}P_{sat\;}}{RT}$$

On the surface of the droplet, the evaporative mass flux of water vapor can be expressed as:

(9) $$J=M_{vapor}\vec{n}\cdot \left(-D\nabla c\right)$$

$\vec{n}$ is the droplet surface normal, and *D* is the diffusion coefficient of water vapor in air. To simplify the calculation, in this paper, the diffusion coefficient of water vapor in air is assumed to be constant and taken as 2.85 × 10^−5^ m^2^/s.

In general, the Marangoni effect occurs when there is a gradient in surface tension at the gas-liquid interface, and surface tension usually changes when the solute concentration, surfactant concentration and temperature along the interface change. In this paper, we focus on the evaporation of droplets under changing temperature conditions; therefore, the change in surface tension σ in the thermally induced Marangoni effect can be described as follows:

(10) $$d\sigma =\frac{\partial \sigma }{\partial T}dT+\frac{\partial \sigma }{\partial c}dc$$

(11) $$\left[\mu \left(\nabla u+{\left(\nabla u\right)}^{T}\right)\right]\cdot \vec{n}=\left|\frac{d\sigma }{dT}\right|\nabla_{t}T$$

### 2.3. Model Validation

The object of study in this article is a droplet of pure water, and under the assumption that evaporation is purely diffusion-driven, the rate of evaporation of a hemispherical droplet can be described as follows [26]:

(12) $$\frac{dV}{dt}=-2\pi RD\frac{\rho_{sat}-\rho_{\infty }}{\rho_{liquid}}$$

where *V* is the droplet volume; *ρ_sat_* is the saturated vapour density; *ρ_∞_* = 0 is the distant vapour density; and *ρ_liquid_* is the droplet density.

**Figure 3:**
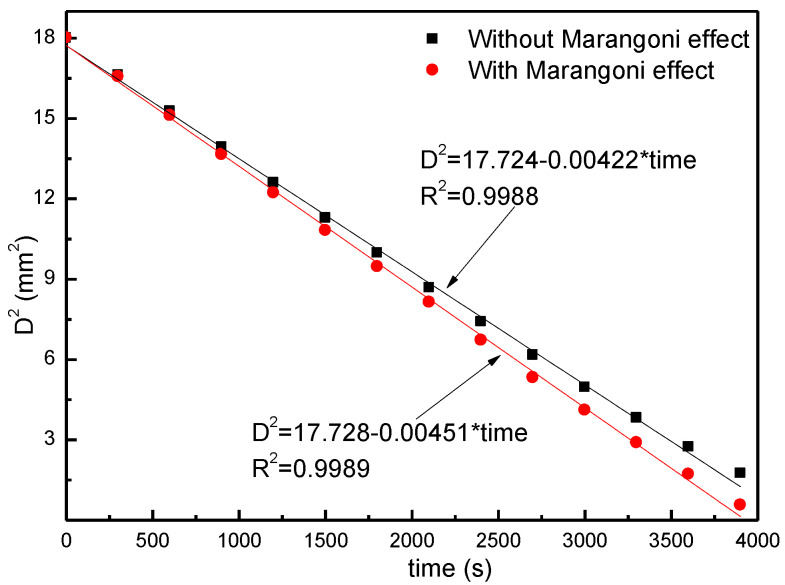
Variation of the squared droplet diameter with evaporation time.

**Figure 4:**
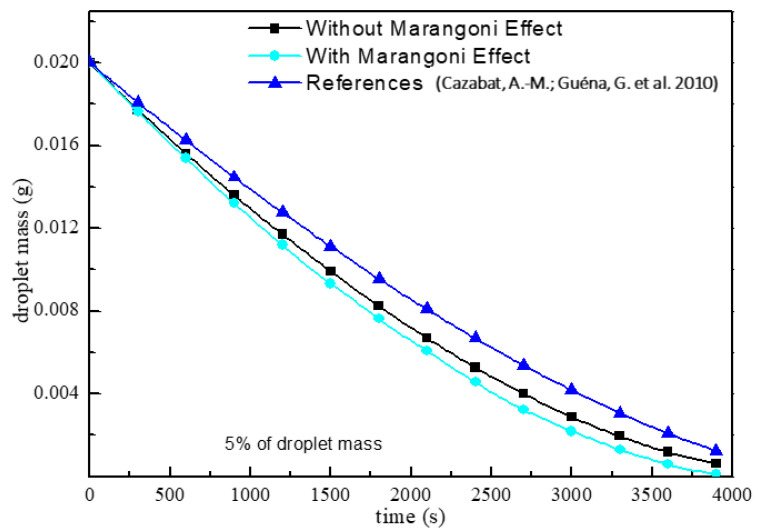
Comparison of simulated values with theoretical values from the literature (Reference data ia from Ref. [26]).

A droplet contact angle of 90 degrees is used, and the law of variation of droplet diameter squared with evaporation time is illustrated in Figure 3. With a linear fit to the data and a correlation coefficient of R^2^ ≈ 1, we can see that the square of the droplet diameter decreases linearly with the evaporation time, and this finding is in agreement with the D^2^ law of classical droplet evaporation [27]. As a further check on the reasonableness of the numerical model above, according to the theoretical droplet evaporation Equation (12) proposed in the literature [26], it is possible to obtain a trend of droplet mass change with time, which is compared to the numerical results in this paper. As can be seen in Figure 4, with continuous evaporation, the evaporation rate of the droplet gradually decreases, and the overall law of change is consistent. With or without the Marangoni effect, when the volume of the droplet is 20 μL with a dry evaporation time near 3870 s, it is near the theoretical evaporation time. On the other hand, when considering the Marangoni effect, the time taken to evaporate at the same droplet radius is shortened slightly, but the two are fundamentally consistent in the law of evaporation. The above numerical model can be seen to be reasonable for investigating the evaporation law of the CCA mode of microdroplets.

## 3. Numerical Results and Analysis

### 3.1. Effect of the Marangoni Effect on the Physical Field Inside the Droplet

**Figure 5:**
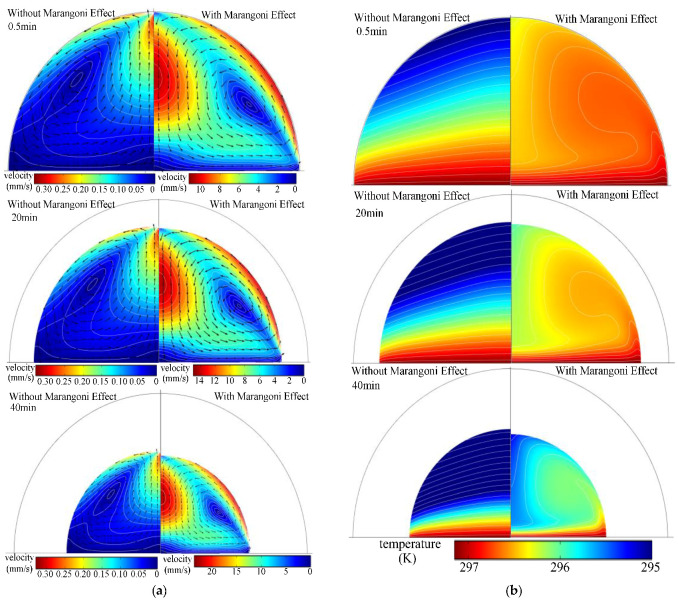
Differences in physical fields within droplets with and without the Marangoni effect. (**a**) Velocity field distribution within the droplet; (**b**) Temperature field distribution within the droplet.

Figure 5 shows the effect of the Marangoni effect on the physical field inside the droplet at an initial droplet volume of 20 μL and an ambient temperature of 297.15 K. Figure 5a shows the distribution of the velocity field inside the droplet. It can be seen that, when there is no Marangoni effect, the direction of flow within the droplet shows a pattern from the bottom centerline to the top of the droplet, which is primarily caused by the evaporative cooling effect at the droplet surface that causes the temperature at the bottom of the droplet to be higher than that at the top of the droplet. This generates a density difference, forming an upward buoyancy force and driving natural convection, and the surface of the droplet forms a center. Due to the small drop size, the temperature difference within the droplet is small, and the natural convection effect is small; thus, the maximum flow velocity is approximately 0.34 mm/s, and the change in flow velocity value as a function of evaporation time is not evident. The rule of thumb is similar to that of Ref. [8]; whereas, when the Marangoni effect is considered, the flow pattern within the droplet is opposite to that without the Marangoni effect. This phenomenon is rarely noticed in the relevant literature. The phenomenon is primarily due to the fact that the temperature at the bottom of the droplet is higher, leading to a lower surface tension, whereas a lower temperature at the top of the droplet leads to a greater surface tension. This leads to the formation of a gradient in surface tension, giving rise to Marangoni flow. Furthermore, the magnitude of the flow velocity increases by almost hundreds of times relative to the absence of the Marangoni effect, and the longer the evaporation time, the greater the value of the flow speed within the droplet. The temperature field distribution within the droplet is shown in Figure 5b. We have shown that, when there is no Marangoni effect, the temperature difference within the droplet is small, generating weak natural convection. That convective heat transfer to the internal heat transfer from the droplet is nearly negligible; thus, the internal temperature of the droplet is partitioned into the heat conduction and heat transfer characteristics of the droplet, and isotherms are near the horizontal and parallel distribution curves. The Marangoni effect reinforces convection within the droplet, causing the overall temperature of the droplet to rise. Simultaneously, the Marangoni effect causes a larger internal flow velocity, which reinforces convective heat transfer. From the formation of the central region, the temperature is low, and the temperature on the outer side is high.

**Figure 6:**
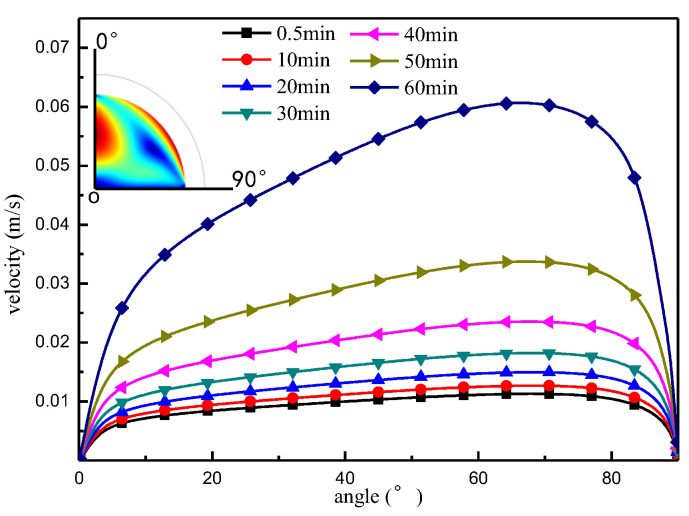
Droplet surface flow rate distribution.

**Figure 7:**
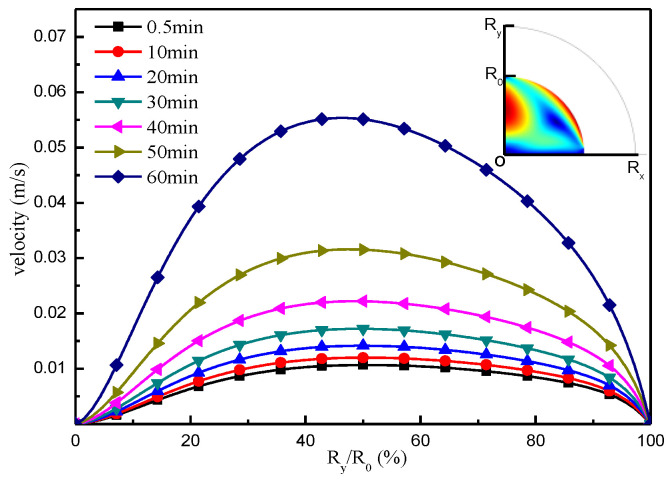
Flow velocity distribution on the droplet centerline.

Figure 6 and Figure 7 show the flow velocity distribution at the boundary of the liquid surface and the centerline of the droplet, respectively, under the Marangoni effect. The change in flow velocity at the liquid surface boundary is shown in Figure 6. From these results, it can be seen that, under the Marangoni effect, the flow velocity from the top to the bottom of the droplet along the liquid surface boundary direction exhibits the law of first increasing and then decreasing. As the evaporation time is extended, the flow speed at the droplet boundary increases continuously, and the high-velocity surface is more concentrated. Figure 7 shows the change in flow velocity along the center of the droplet, which is influenced by the Marangoni effect.

**Figure 8:**
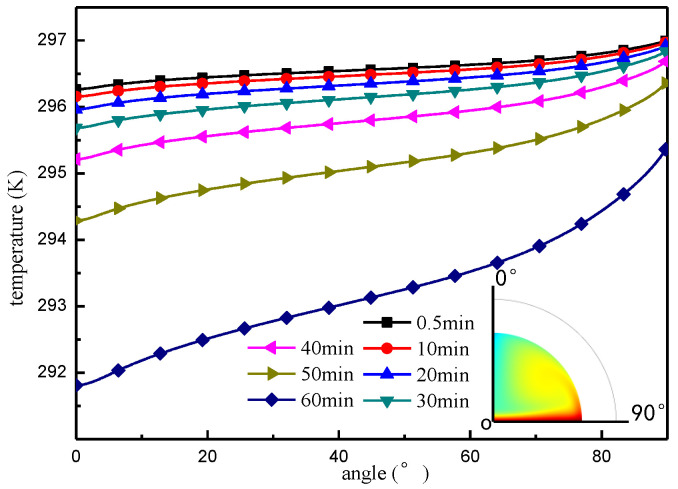
Droplet surface temperature distribution.

**Figure 9:**
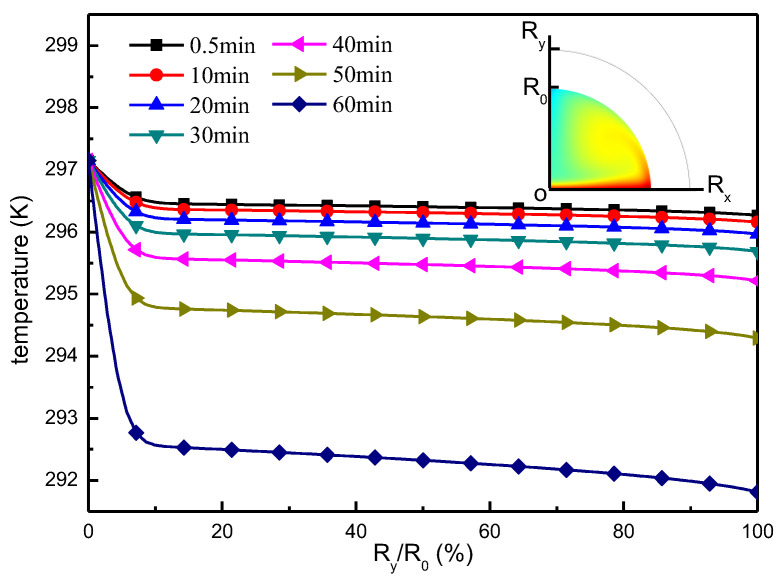
Temperature distribution on the centerline of the droplet.

The temperature distributions on the liquid surface boundary and droplet centerline, under the Marangoni effect, are shown in Figure 8 and Figure 9, respectively. From the results in Figure 8 we see that, at the onset of evaporation, there is a relatively small difference in temperature between the top and bottom of the droplet. As evaporation progresses, the temperature of the top and the bottom of the droplet become a decreasing trend, and the temperature difference becomes progressively larger with a greater drop in temperature from the top of the droplet. The temperature distribution on the droplet centerline is shown in Figure 9, and the results demonstrate that the temperature of the droplet close to the substrate is higher, but along the centerline upward, the temperature remains fundamentally at a stable low temperature level. As evaporation develops, the temperature in the low-temperature region decreases in an increasingly obvious way.

**Figure 10:**
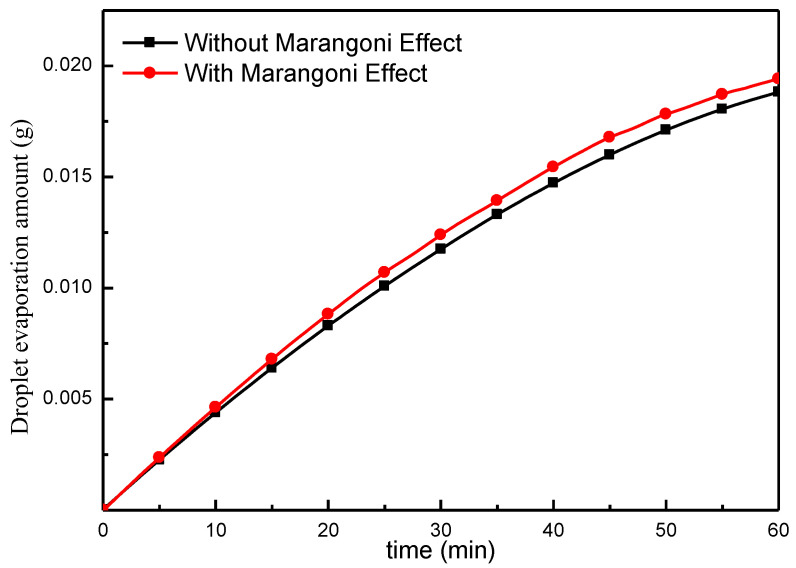
Effect of the presence or absence of Marangoni benefits on evaporation quality.

As shown in Figure 5, without the Marangoni effect, convection within the droplet is weak, and the heat transfer is dominated by the heat conduction of the droplet. In order to further compare the contribution of the Marangoni effect to the efficiency of evaporation, in order to quantify and compare the two cases, we use the evaporation mass change of the droplet, as can be seen in Figure 10. The results show that, compared to the lack of the Marangoni effect, the effect can be used to improve the efficiency of evaporation, combined with the change in temperature and velocity fields between. The longer the duration of evaporation, the larger the temperature difference between the liquid at the top of the droplet and in the vicinity of the substrate, causing the Marangoni flow to be more intense and thus strengthening the convective heat exchange process of the droplets. The rate of evaporation is increased by almost 2.5%, compared to no Marangoni benefit. From Equation (9) one can see that, with the development of evaporation, the droplet surface area gradually decreases, and the evaporation rate is gradually reduced.

### 3.2. Effect of Droplet Initial Radius on Droplet Evaporation

**Figure 11:**
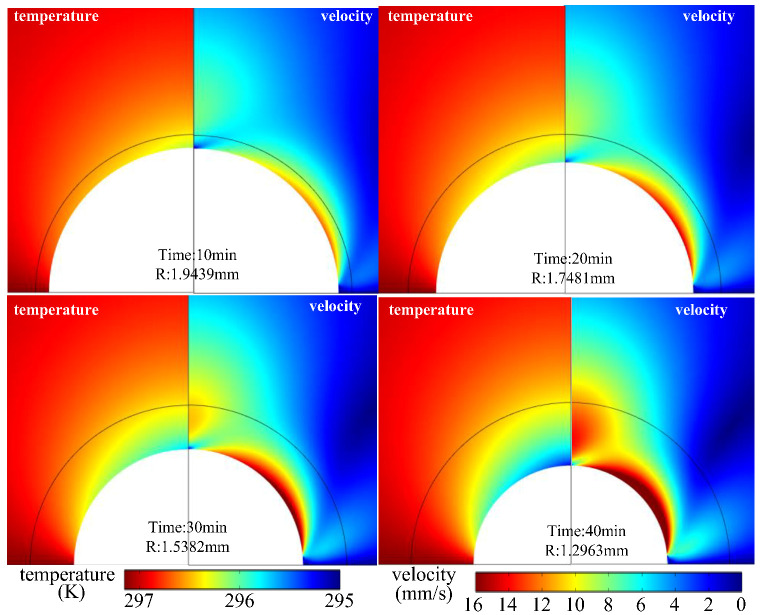
Distribution of temperature and velocity fields near the droplet surface as the droplet evaporates to different droplet radii.

The change in droplet radius and temperature and velocity distribution in the air domain close to the droplet during evaporation of the same droplet are shown in Figure 11. This shows that, as the vaporization process of water is a process of heat absorption, there is a decrease in air temperature near the droplet surface. In particular, the air temperature at the top of the droplet decreases in a more obvious way the greater the temperature decrease.

**Figure 12:**
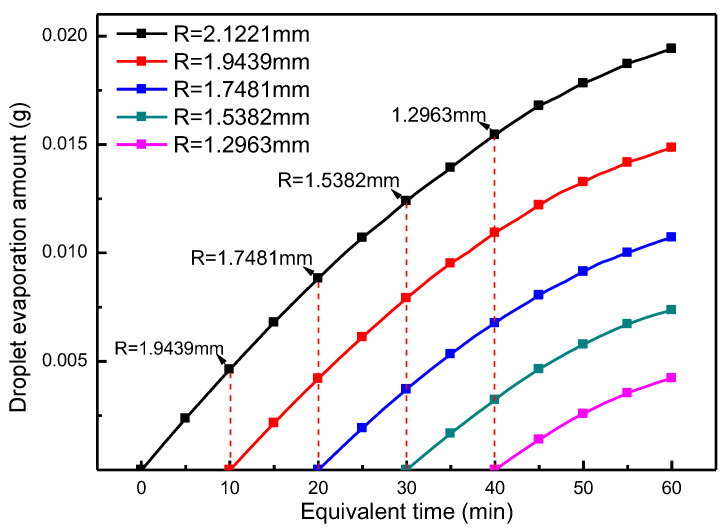
Evaporation at each moment for different initial droplet radii.

The law of evaporation states that the velocity of the airflow on the droplet surface can also affect the evaporation process of the droplet. Figure 11 shows that the evaporation of droplets increases the air flow rate at the liquid surface, and as evaporation proceeds, the air flow rate near the liquid surface also increases; this means that this process is likely to be a self-reinforcing evaporation process. In order to analyze this question, this section compares the evaporation of droplets with different initial radii selected. Figure 11, at each instant corresponding to the initial radius of the droplet, provides a good comparison with the evaporation onset time for each droplet at the time corresponding to the chosen droplet radius. Because of the close variation in evaporation capacity during evaporation of droplets with different initial radii, to make the law more clear, we introduce the equivalent time as an abscissa. If the amount of evaporation is 0, the corresponding equivalent time corresponds to the initial evaporation time. Before this, the droplet is assumed not to evaporate. The results in Figure 12 show that the process of evaporation change of different initial radius of the droplet fundamentally coincides; essentially, the characteristic time corresponding to droplet diameter is the same. Thus, the initial radius of the droplet has little influence on the law of evaporation. This demonstrates that the initial droplet radius has little effect on the law of evaporation, and the increase in air velocity close to the liquid surface caused by evaporation does not have an obvious effect on the increased evaporation. The process of droplet evaporation is, thus, not a self-improving process.

### 3.3. Effect of Ambient Temperature on Droplet Evaporation

**Figure 13:**
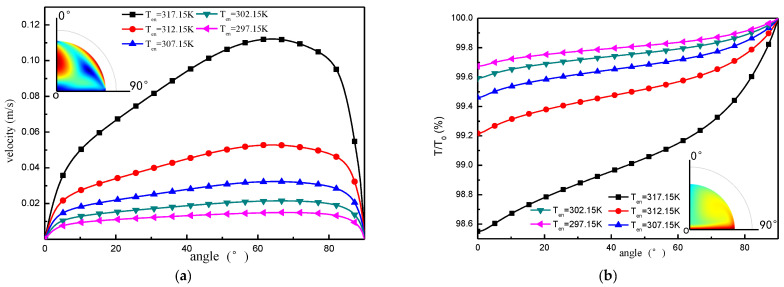
Variation of the physical field on the liquid surface boundary line at different ambient temperatures. (**a**) Variation in droplet surface flow rate (20 min); (**b**) Change in droplet surface temperature (20 min).

One of the main factors affecting evaporation of liquids is the ambient temperature, whereas according to Equations (6)–(11), the density and surface tension of the droplet are related to the ambient temperature. To analyze the effect of the ambient temperature on the temperature and velocity distribution inside the droplet due to the Marangoni effect, in this section, the ambient temperatures of 297.15 K, 302.15 K, 307.15 K, 312.15 K, and, respectively, 317.15 K were compared to the changes in the flow velocity and temperature of the liquid surface during the evaporation of the 20 μL droplets, as shown in Figure 12. This shows that increasing the ambient temperature strengthens the Marangoni flow, and the surface liquid flow velocity increases. The increase in flow velocity is nonlinearly related to ambient temperature; the higher the temperature, the larger the increase in flow. So that the difference in the change in liquid surface temperature at different ambient temperatures could be compared, the ratio of the temperature to the ambient temperature at each part of the liquid surface will be used as an index of comparison. The results in Figure 13a show that, as the ambient temperature increases, the temperature difference at the liquid surface becomes more extreme. (The temperature at the top of the droplet is a very small value, and the temperature at the bottom edge of the liquid surface is an extreme value).

**Figure 14:**
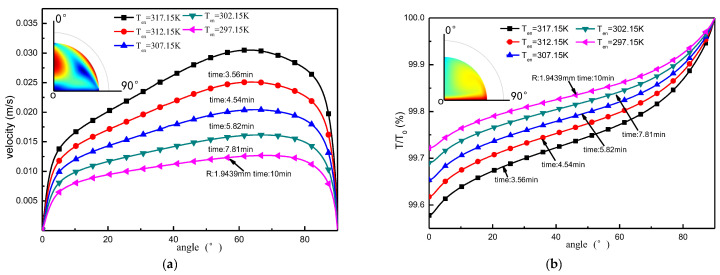
Variation of the physical field on the liquid surface boundary line for different ambient temperature conditions when the droplets evaporate to the same radius. (**a**) Variation in droplet surface flow rate; (**b**) Change in droplet surface temperature.

To further analyze the liquid surface flow rate and temperature change process during the evaporation of droplets, a 20 μL droplet evaporated to a droplet radius of 1.9439 mm was chosen as the baseline for comparison in this section, and the results of the comparison are shown in Figure 14. As the temperature is increased linearly, the results show that the time taken to evaporate to the specified droplet radius exhibits nonlinear shortening, but the increase in flow velocity and temperature at each point on the liquid surface exhibits a quasi-linear increasing profile.

**Figure 15:**
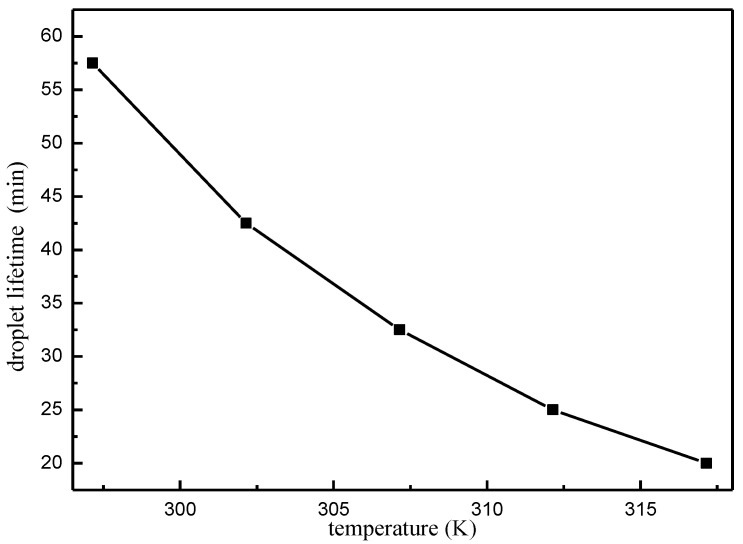
Droplet life versus ambient temperature variation curve.

Feargus [28] investigated the process of droplet evaporation on the thermally conducting substrate by constructing a coupling model and ignoring the Marangoni effect, emphasizing that the initial contact angle and substrate temperature will both affect the lifetime of the droplet. This paper considers the Marangoni effect and considers environmental temperature as a variable for extensive research, and the curve between the droplet lifetime and the environmental temperature is obtained as shown in Figure 15. It can be seen that, when the temperature is on the order of 297.15 K to 317.15 K, there is a nonlinear relationship between droplet lifetime (time required for evaporation) and ambient temperature; the higher the temperature, the shorter the droplet lifetime, but the higher the temperature, the lower the rate of droplet lifetime decay.

**Figure 16:**
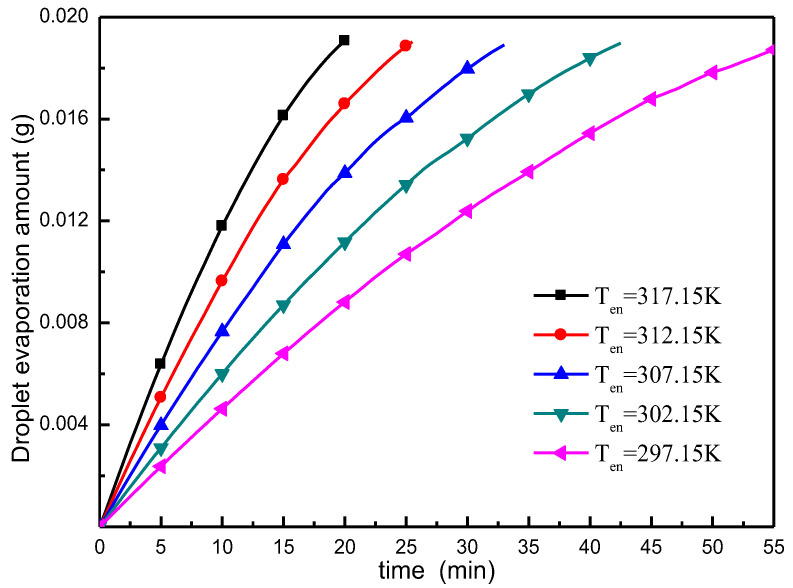
Variation in droplet evaporation mass at different ambient temperatures.

The variation of droplet evaporative mass with time at different ambient temperatures is shown in Figure 16. This shows that an increase in the ambient temperature can significantly increase the rate of evaporation. At different ambient temperatures, the same radius of the droplet evaporates at the same time, and the relationship between increasing evaporation quality of droplets and increasing temperature is nonlinear. For each 5 °C increase in ambient temperature, the maximum increase in the rate of evaporation is approximately 22.7%.

## 4. Conclusions

The influence of the Marangoni effect on droplet heat and mass transfer during droplet evaporation is primarily investigated in this paper. The next step will be to modulate the Marangoni effect by surfactant or by adding external physical fields, laying the foundation for achieving precise control of the evaporation process.

In this paper, through the construction of a numerical thermal mass coupling model for droplet evaporation and the verification of the accuracy of the model via theoretical values in the reference literature, we investigate the characteristics of the droplet flow field and the temperature field distribution at the gas–liquid interface during evaporation of tiny droplets on the wall. The main conclusions that can be drawn from the study are as follows:

(1) When considering the Marangoni effect, the evaporation process of the wall droplet leads to a slight increase in the bulk temperature of the droplet, and generation of Marangoni flow may increase the internal droplet velocity. In comparison to the lack of the Marangoni effect, the effect can increase the internal velocity of the droplet by almost 100 times and increase the rate of evaporation by approximately 2.5%.
(2) In the evaporative regime, the air temperature near the liquid surface exhibits a decreasing profile, and the flow velocity inside the air domain increases. However, increasing the rate of air flow at the liquid surface by evaporation is not enough to further increase droplet evaporation.
(3) Ambient temperatures can reinforce Marangoni flow and increase liquid surface velocity and temperature, shortening the droplet drying time; there is a non-linear variation between increasing the evaporation quality of the droplet and increasing the temperature of the droplet. For each 5 °C increase in ambient temperature, the maximum increase in the rate of evaporation is approximately 22.7%.
